# Upregulation of the heterogeneous nuclear ribonucleoprotein hnRNPA1 is an independent predictor of early biochemical recurrence in TMPRSS2:ERG fusion-negative prostate cancers

**DOI:** 10.1007/s00428-020-02834-4

**Published:** 2020-05-16

**Authors:** Katharina Möller, Anna Lena Wecker, Doris Höflmayer, Christoph Fraune, Georgia Makrypidi-Fraune, Claudia Hube-Magg, Martina Kluth, Stefan Steurer, Till S. Clauditz, Waldemar Wilczak, Ronald Simon, Guido Sauter, Hartwig Huland, Hans Heinzer, Alexander Haese, Thorsten Schlomm, Sören Weidemann, Andreas M. Luebke, Sarah Minner, Christian Bernreuther, Sarah Bonk, Andreas Marx

**Affiliations:** 1 Institute of Pathology, University Medical Center Hamburg-Eppendorf, Martinistr. 52, 20246 Hamburg, Germany; 2 Martini-Clinic, Prostate Cancer Center, University Medical Center Hamburg-Eppendorf, Hamburg, Germany; 3 Department of Urology, Charité - Universitätsmedizin Berlin, Berlin, Germany; 4 General, Visceral and Thoracic Surgery Department and Clinic, University Medical Center Hamburg-Eppendorf, Hamburg, Germany; 5 Institute of Pathology, Klinikum Fürth, Fürth, Germany

**Keywords:** hnRNPA1, Prognosis, Prostate cancer, TMA

## Abstract

Heterogeneous nuclear ribonucleoprotein A1 (hnRNPA1) is a ubiquitous RNA splicing factor that is overexpressed and prognostically relevant in various human cancer types. To study the impact of hnRNPA1 expression in prostate cancer, we analyzed a tissue microarray containing 17,747 clinical prostate cancer specimens by immunohistochemistry. hnRNPA1 was expressed in normal prostate glandular cells but often overexpressed in cancer cells. hnRNPA1 immunostaining was interpretable in 14,258 cancers and considered strong in 33.4%, moderate in 45.9%, weak in 15.3%, and negative in 5.4%. Moderate to strong hnRNPA1 immunostaining was strongly linked to adverse tumor features including high classical and quantitative Gleason score, lymph node metastasis, advanced tumor stage, positive surgical margin, and early biochemical recurrence (*p* < 0.0001 each). The prognostic impact of hnRNPA1 immunostaining was independent of established preoperatively or postoperatively available prognostic parameters (*p* < 0.0001). Subset analyses revealed that all these associations were strongly driven by the fraction of cancers lacking the TMPRSS2:ERG gene fusion. Comparison with other key molecular data that were earlier obtained on the same TMA showed that hnRNPA1 overexpression was linked to high levels of androgen receptor (AR) expression (*p* < 0.0001) as well as presence of 9 of 11 chromosomal deletions (*p* < 0.05 each). A strong association between hnRNPA1 upregulation and tumor cell proliferation that was independent from the Gleason score supports a role for tumor cell aggressiveness. In conclusion, hnRNPA1 overexpression is an independent predictor of poor prognosis in ERG-negative prostate cancer. hnRNPA1 measurement, either alone or in combination, might provide prognostic information in ERG-negative prostate cancer.

**Electronic supplementary material:**

The online version of this article (10.1007/s00428-020-02834-4) contains supplementary material, which is available to authorized users.

## Introduction

Prostate cancer is the most prevalent cancer in men in Western societies [46]. Despite a rather indolent clinical course of most prostate cancers, this disease still represents the third most common cause of cancer-related death in men. A reliable distinction between indolent and aggressive forms of the disease is highly desirable to improve therapeutic decision-making. The only established pretreatment prognostic parameters currently include Gleason score and tumor extent on biopsies, preoperative prostate-specific antigen (PSA) level, and clinical stage. There is hope that a deeper insight in disease biology will eventually identify clinically applicable molecular markers that implicate a more reliable prediction of prostate cancer aggressiveness.

Heterogeneous nuclear ribonucleoprotein A1 (hnRNPA1) is the most abundantly expressed member of a family of more than 20 related proteins. hnRNPs form “beads on a string” like highly dynamic complexes that bind nascent nuclear RNAs and regulate their transcription, splicing, stability, export from the nucleus, and translation [41]. hnRNPA1-dependent alternative splicing affects many genes related to growth signaling and DNA repair, including for example SRC kinase, the HRAS oncogene, and the BRCA1 tumor suppressor [17]. In addition, hnRNPA1 has been reported to bind to telomeric sequences where it stimulates telomerase activity and contributes to telomere length regulation and maintenance [41]. It is, thus, not surprising that deregulation—typically overexpression—of hnRNPA1 has been linked to a variety of diseases including cancer. For example, overexpression of hnRNPA1 has been reported from gliomas, lymphomas, myelomas, leukemias, and breast, colorectal, gastric, and lung cancers [2, 4, 7, 16, 38, 44, 47], and was linked to poor prognosis in hepatocellular carcinoma [50] and breast cancer [37]. There is growing evidence that hnRNPA1 also plays an important role in prostate cancer biology and therapy. hnRNPA1 regulates expression of androgen receptor splice variants and plays a role in enzalutamide sensitivity [36]. However, data on the prevalence and clinical significance of hnRNPA1 protein expression in prostate cancer are still lacking in the literature.

Here, we took advantage of our large set of more than 17,000 cancer specimens available in a tissue microarray (TMA) format to study hnRNPA1 protein expression in prostate cancer and to determine its clinical significance.

## Materials and methods

### Patients

Radical prostatectomy specimens were available from 17,747 patients, undergoing surgery between 1992 and 2015 at the Department of Urology and the Martini Clinics at the University Medical Center Hamburg-Eppendorf. All prostate specimens were analyzed according to a standard procedure, including a complete embedding of the entire prostate for histological analysis [43]. Prostate cancer staging was performed according to the guidelines of the American Joint Committee on Cancer 2016. Histopathological data were retrieved from the patients’ records, including tumor stage, Gleason score, nodal stage, and stage of the resection margin. In addition to the classical Gleason categories, “quantitative” Gleason grading was performed as described before [42]. In brief, for every prostatectomy specimen, the percentages of Gleason 3, 4, and 5 patterns were recorded. Gleason 7 cancers were subdivided in 7 subgroups according to their percentage of Gleason 4: 3 + 4 ≤ 5% Gleason 4, 3 + 4 6–10%, 3 + 4 11–20%, 3 + 4 21–30%, 3 + 4 31–49%, 4 + 3 50–60%, and 4 + 3 > 60% Gleason 4. Additional groups were defined by the presence of a tertiary Gleason 5 pattern, including 3 + 4 Tert.5 and 4 + 3 Tert.5. Follow-up data were available for a total of 14,664 patients with a median follow-up of 48 months (range, 1 to 275 months; Table 1). Prostate-specific antigen (PSA) values were measured following surgery and PSA recurrence was defined as a postoperative PSA of ≥ 0.2 ng/ml or increasing PSA values in subsequent measurements. The TMA manufacturing process was described earlier in detail [27, 34]. In short, one 0.6-mm core was taken from a tumor containing tissue block from each patient. The tissues were distributed among 39 TMA blocks. For internal controls, each TMA block also contained various control tissues, including normal prostate tissue. The molecular database included data on KI67 labeling index (Ki67LI) from 5492 tumors (expanded from [45]), ERG protein expression from 13,089 and ERG rearrangement analysis by fluorescence in situ hybridization (FISH) from 7225 tumors [32, 33], as well as deletion status of 3p13 (*FOXP1*) from 5503 tumors (expanded from [29]), 5q21 (*CHD1*) from 6145 tumors (expanded from [6]), 6q15 (*MAP 3K7*) from 4663 tumors (expanded from [20]), 8p21 from 5556 tumors [24], 10q23 (*PTEN*) from 5158 tumors (expanded from [28]), 12p13 (*CDKN1B*) from 4887 tumors [22], 12q24 from 5721 tumors [49], 13q14 (*ENOX1*) from 5915 tumors [26], 16q24 from 4413 tumors [25], 17p13 (TP53) from 6437 tumors (expanded from [21]), and 18q21 from 5578 tumors [23]. The usage of archived diagnostic left-over tissues for manufacturing of tissue microarrays and their analysis for research purposes as well as patient data analysis has been approved by local laws (HmbKHG, §12,1) and by the local ethics committee (Ethics Commission Hamburg, WF-049/09). All work has been carried out in compliance with the Helsinki Declaration.

**Table 1:** Pathological and clinical data of the arrayed prostate cancers

	No. of patients (%)	
	Study cohort on TMA* (*n* = 17,747)	Biochemical relapse among categories**
Follow-up (month)		
*n*	14,464 (81.5%)	3612 (25%)
Mean	56.3	–
Median	48	–
Age (year)		
≤ 50	433 (2.4%)	66 (15.2%)
51–59	4341 (24.5%)	839 (19.3%)
60–69	9977 (56.4%)	2073 (20.8%)
≥ 70	2936 (16.6%)	634 (21.6%)
Pretreatment PSA (ng/ml)		
< 4	2225 (12.6%)	313 (14.1%)
4–10	10,520 (59.6%)	1696 (16.1%)
10–20	3662 (20.8%)	1043 (28.5%)
> 20	1231 (7%)	545 (44.3%)
pT stage (AJCC 2016)		
pT2	11,518 (65.2%)	1212 (10.5%)
pT3a	3842 (21.7%)	1121 (29.2%)
pT3b	2233 (12.6%)	1213 (54.3%)
pT4	85 (0.5%)	63 (74.1%)
Gleason grade		
≤ 3 + 3	3570 (20.3%)	264 (7.4%)
3 + 4	9336 (53%)	1436 (15.4%)
3 + 4 Tert.5	798 (4.5%)	165 (20.7%)
4 + 3	1733 (9.8%)	683 (39.4%)
4 + 3 Tert.5	1187 (6.7%)	487 (41%)
≥ 4 + 4	999 (5.7%)	531 (53.2%)
pN stage		
pN0	10,636 (89.4%)	2243 (21.1%)
pN+	1255 (10.6%)	700 (55.8%)
Surgical margin		
Negative	14,297 (80.8%)	2307 (16.1%)
Positive	3388 (19.2%)	1304 (38.5%)

*Percentage refers to the fraction of samples across each category. **Percentage refers to the fraction of samples with biochemical relapse within each parameter in the different categories

### Immunohistochemistry

Freshly cut TMA sections were immunostained on one day and in one experiment. Slides were deparaffinized and exposed to heat-induced antigen retrieval for 5 min in an autoclave at 121 °C in pH 7.8 Tris-EDTA-Citrate buffer. Primary antibody specific for hnRNPA1 (mouse monoclonal, clone 9H10, Abcam cat. no. ab5832; dilution 1:4050) was applied at 37 °C for 60 min. Bound antibody was then visualized using the EnVision Kit (Dako, Glostrup, Denmark) according to the manufacturer’s directions. hnRNPA1 staining was nuclear and typically affected all (100%) cells in a tissue spot, including cancerous and non-cancerous cells. Only tumor cells were scored. Tumors with complete absence of staining were scored as “negative.” All other tumors were graded according to the staining intensity as “weak,” “moderate,” or “strong.”

### Statistics

For statistical analysis, the JMP 12.0 software (SAS Institute Inc., NC, USA) was used. Contingency tables were calculated to study association between hnRNPA1 expression and clinico-pathological variables, and the chi-square (likelihood) test was used to find significant relationships. Analysis of variance (ANOVA) and *F*-test was applied to find associations between hnRNPA1 expression and tumor cell proliferation as measured by Ki67LI. Kaplan Meier curves were generated using biochemical (PSA) recurrence as the clinical end point. The log-rank test was applied to test the significance of differences between stratified survival functions. Cox proportional hazards regression analysis was performed to test the statistical independence and significance between pathological, molecular, and clinical variables.

## Results

### Technical issues

A total of 14,258 (80%) tumor samples were interpretable in our TMA analysis. Reasons for non-informative cases (3489 spots; 20%) included lack of tissue samples or absence of unequivocal cancer tissue in the TMA spots (Table 1).

### hnRNPA1 expression in normal and cancerous prostate tissue

**Fig. 1:**
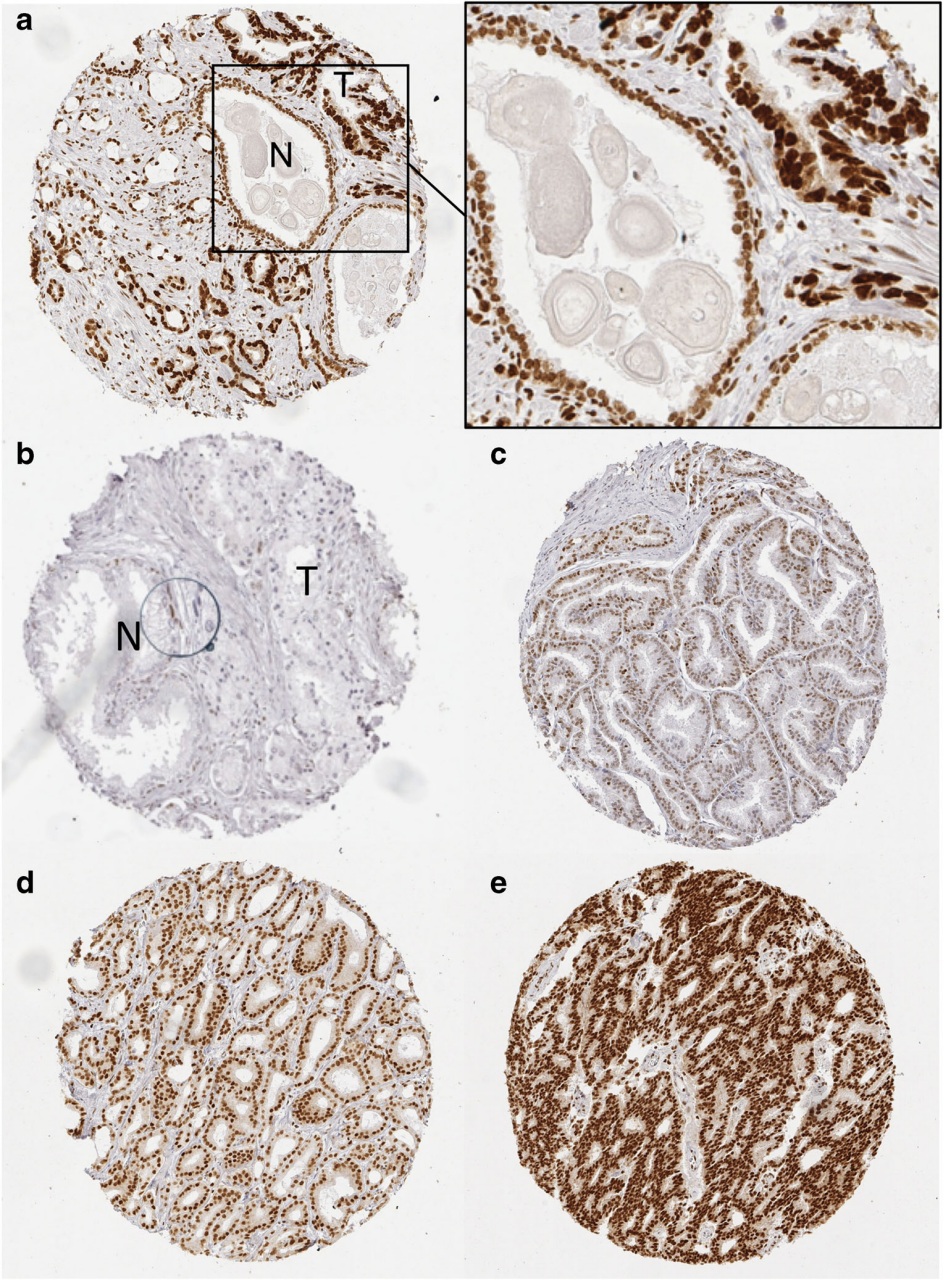
Representative pictures of hnRNPA1 immunostaining in prostate cancers. (a) Tissue spot containing both normal (N) and tumor (T) glands. The magnification shows that tumor glands overexpress hnRNPA1 in comparison to normal glands. (b) Tissue spots with tumor glands (T) lacking hnRNPA1 staining. Adjacent normal glands (N) are also negative except from some faint staining of basal cells. (c) Weak, (d) moderate, (e) strong staining

Normal prostate glands showed strong nuclear staining of basal cells and less intense (i.e., weak to moderate) staining of luminal cells. Weak to moderate staining was also seen in stroma cells. Cancer cells typically stained moderately (45.9%) to strongly (33.4%) positive. Weak staining was seen in 15.3% of cancers. Negative staining was rare (5.4%) and was always associated by a complete lack of staining in all (tumor and normal) cells. Direct comparison of normal and cancer glands in the same tissue spot revealed that cancer glands typically stained stronger than adjacent normal glands. Representative images of hnRNPA1 immunostainings are given (Fig. 1).

### hnRNPA1 and *TMPRSS2:ERG* fusion status

The *TMPRSS2:ERG* status was available from 6196 cancers (using ERG break apart FISH analysis) and from 11,521 cancers (using ERG-IHC) of all tumors with interpretable data on hnRNPA1. There were 5142 ERG-negative and 4441 ERG-positive cancers by ERG-IHC analysis, as well as 3393 ERG-negative and 2803 ERG-positive cancers by ERG-FISH analysis. ERG-FISH and ERG-IHC showed highly concordant results: an identical finding (ERG-IHC positive and break by FISH or ERG-IHC negative and missing break by FISH) was found in 5256 of 5582 (94%) cancers. hnRNPA1 upregulation was more frequent (*p* < 0.0001) in ERG-positive than in ERG-negative cancers: Strong immunostaining was seen in 41.0% of cancers with ERG-IHC-positive staining and 45% of cancers with ERG rearrangements, but only in 22% of ERG-IHC-negative cancers and 29% of cancers without TMPRSS2:ERG fusion detected by FISH (*p* < 0.0001 each; Supplementary Fig. 1). Due to these differences, we performed all further statistical analyses also in subsets of ERG-negative and ERG-positive cancers.

### hnRNPA1 and tumor phenotype

Moderate to strong immunostaining was significantly linked to advanced tumor stage, high Gleason score, lymph node metastasis, high preoperative PSA level (*p* < 0.0001 each), and tumor-positive resection margin (*p* = 0.0033; Table 2). Additional subset analyses were performed because of the strong association between hnRNPA1 and *TMPRSS2:ERG* fusion. It showed that most of these associations were driven by the subset of ERG-negative cancers (Supplementary Table 1) but failed to reach statistical significance in ERG-positive cancers (Supplementary Table 2).

**Table 2:** hnRNPA1 staining and prostate cancer phenotype

		hnRNPA1 result					
		*n* evaluable	Negative (%)	Weak (%)	Moderate (%)	Strong (%)	*p* value
All cancers		14,258	5.4	15.3	45.9	33.4	
Tumor stage	pT2	9142	6.2	17.3	43.6	32.9	< 0.0001
	pT3a	3132	4.6	12.7	48.9	33.8	
	pT3b-4	1926	2.7	10	51.3	36	
Gleason grade	≤ 3 + 3	2853	8.6	19.6	36.6	35.2	< 0.0001
	3 + 4	7492	5.4	15.9	45.6	33.1	
	3 + 4 Tert.5	647	3.4	14.5	56.1	26	
	4 + 3	1397	3.6	11.3	49.5	35.6	
	4 + 3 Tert.5	958	2.2	9.1	55.7	33	
	≥ 4 + 4	801	2.4	10	55.7	32	
Quantitative Gleason	3 + 4 ≤ 5%	1956	6.7	16.8	43.8	32.7	< 0.0001
	3 + 4 6–10%	1862	5.9	17.3	44.3	32.5	
	3 + 4 11–20%	1617	4.9	14.6	49	31.5	
	3 + 4 21–30%	833	4.4	15.4	45.4	34.8	
	3 + 4 31–49%	678	5.2	12.2	46.9	35.7	
	4 + 3 50–60%	570	3.4	14.5	56.1	26	
	4 + 3 61–80%	492	3.7	11.8	50.4	34.2	
	4 + 3 > 80%	133	3.3	11.6	50.4	34.8	
Lymph node metastasis	N0	8515	4.9	14	47.5	33.7	< 0.0001
	N+	1019	2.4	8.9	55.8	32.9	
Preop. PSA level (ng/ml)	< 4	1762	3.5	12	44.7	39.8	< 0.0001
	4–10	8413	5.3	15.5	44.9	34.3	
	11–20	2972	6.6	16.1	47.7	29.6	
	> 20	1020	5.9	17.2	50.5	26.5	
Surgical margin	negative	11,362	5.4	15.7	45.1	33.7	0.0033
	positive	2843	5.2	13.7	48.7	32.5	

### hnRNPA1 and genomic deletions

**Fig. 2:**
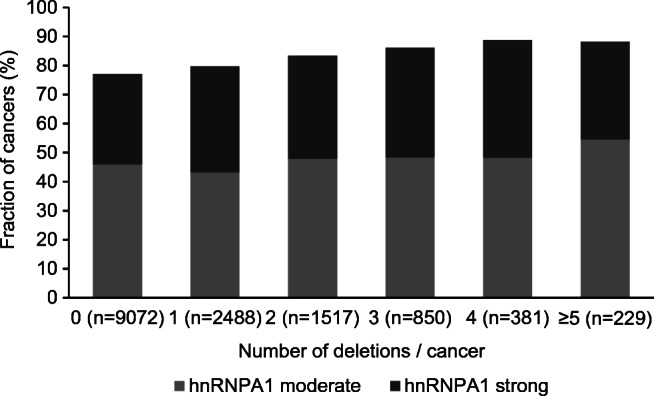
Correlation between hnRNPA1 immunostaining and numbers of deletions in all prostate cancers

Most chromosomal deletions in prostate cancer are linked either to cancers harboring ERG fusions (3p, 8p, PTEN, 12q, 16q, 17p) [21, 24, 25, 28, 29, 49] or to the ERG-negative subset (5q, 6q, 13q, 18q) [6, 20, 23, 26]. Given that hnRNPA1 was strongly associated with ERG-positive cancers itself, it was expected that hnRNPA1 was positively linked to all ERG-associated deletions and inversely linked to all deletions associated to ERG-negative cancers. However, subset analyses revealed that hnRNPA1 overexpression was associated with 9 of 11 deletions in ERG-negative cancers (*p* < 0.05) and with 6 of 11 deletions in ERG-positive cancers (*p* < 0.05, Supplementary Fig. 2). Moderate to strong hnRNPA1 immunostaining was also linked with higher numbers of deletions present in a cancer (Fig. 2).

### hnRNPA1 and tumor cell proliferation (Ki67 labeling index)

hnRNPA1 upregulation was linked to cell proliferation as measured by Ki67LI. The average Ki67LI increased from 1.06 ± 0.1 in cancers with lacking hnRNPA1 expression to 3.15 ± 0.06 in cancers with strong hnRNPA1 (*p* < 0.0001). This association held true in tumor subsets with identical Gleason score (Supplementary Table 3).

### hnRNPA1 and androgen receptor

Immunohistochemical androgen receptor (AR) data were available from a previous study [49]. Data on hnRNPA1 and AR expression were available from 7157 cancers. hnRNPA1 staining was strongly linked to AR levels independently from the ERG status (*p* < 0.0001, Supplementary Fig. 3).

### hnRNPA1 and PSA recurrence

**Fig. 3:**
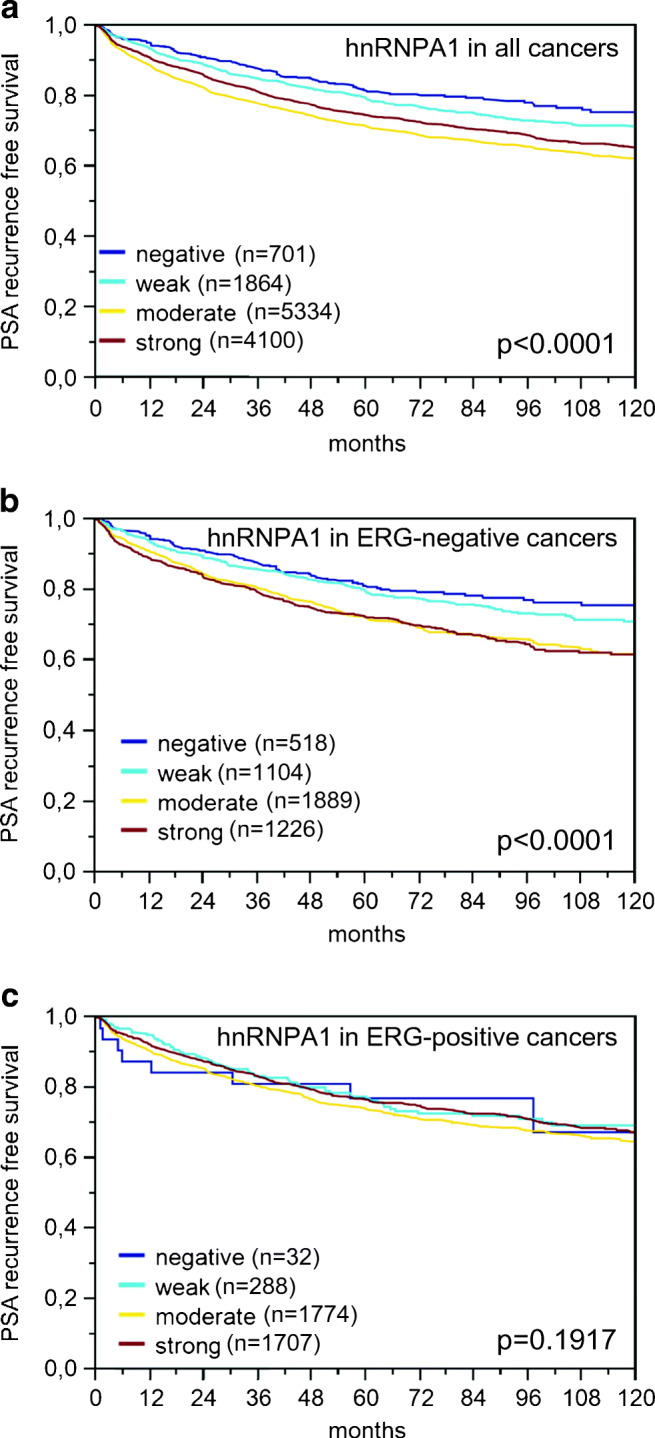
**a**–**c** Prognostic relevance of hnRNPA1

**Fig. 4:**
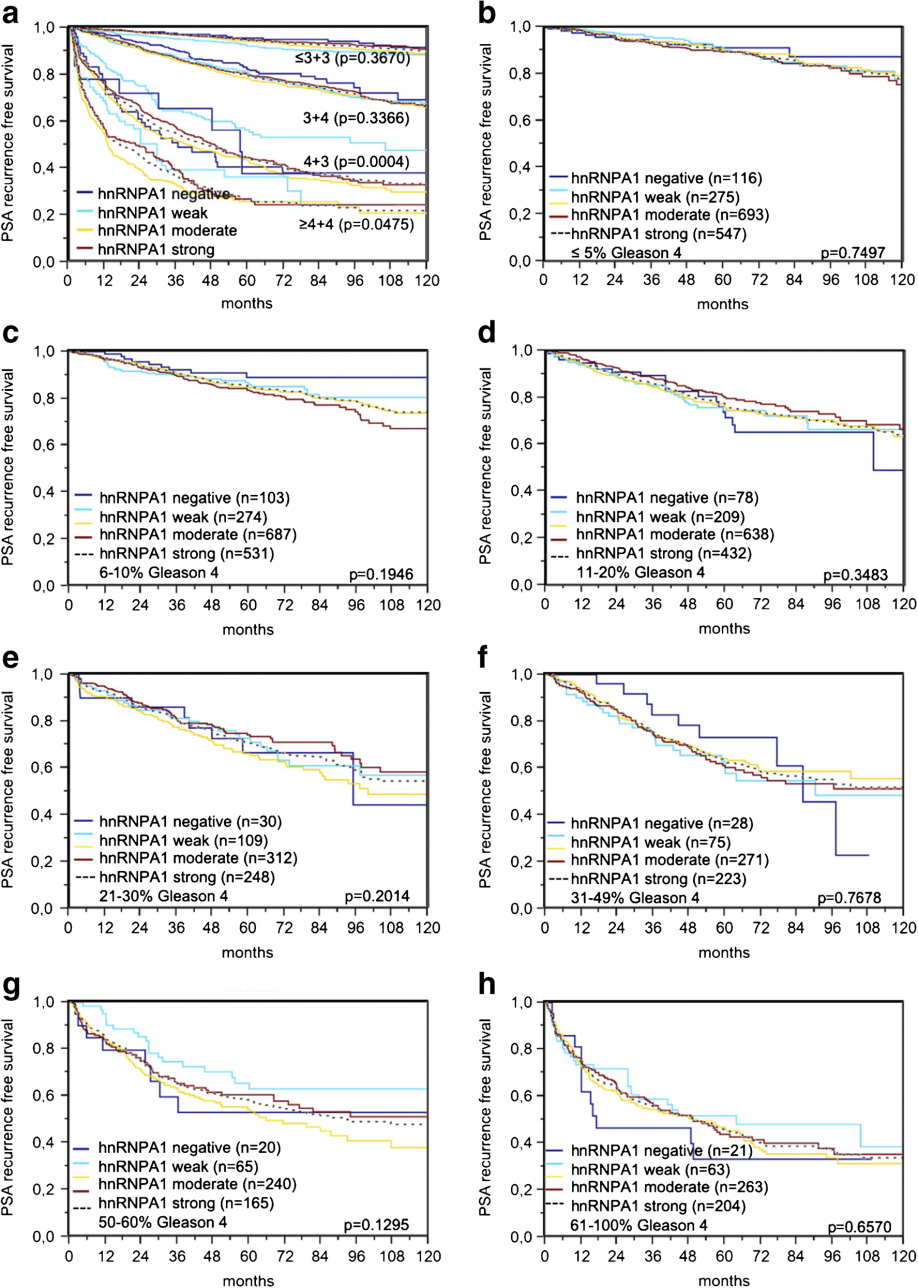
Prognostic relevance of hnRNPA1 immunostaining in **a** cancers with identical classical Gleason score and in **b**–**h** subsets of cancers defined by the same quantitative Gleason score

Increased hnRNPA1 staining was significantly associated with early PSA recurrence (*p* < 0.0001, Fig. 3a). Subset analyses showed that this association was solely driven by the subset of ERG-negative cancers (*p* < 0.0001, Fig. 3b) while outcome differences were not seen in ERG-positive cancers (*p* = 0.1917, Fig. 3c). However, extended analyses in subsets of cancers defined identical classical and quantitative Gleason scores further revealed that hnRNPA1 provided additional prognostic information in Gleason 4 + 3 cancers (*p* = 0.0004, Fig. 4a) but not in cancers with an identical quantitative Gleason score (Fig. 4b–h).

### Multivariate analyses

Four different multivariate analyses were performed to evaluate the clinical relevance of hnRNPA1 expression in different scenarios (Table 3). Scenario 1 evaluated hnRNPA1 and all postoperatively available parameters including pT, pN, surgical margin status, preoperative PSA value, and Gleason score obtained on the prostatectomy specimen. In scenario 2, hnRNPA1 and all postoperatively available parameters except pN were included. The rationale for this approach was that the indication and extent of lymph node dissection is not standardized in the surgical therapy of prostate cancer and may introduce a bias towards high-grade cancers. Two additional scenarios were evaluated to model the preoperative situation as much as possible. Scenario 3 included hnRNPA1 expression, preoperative PSA, clinical tumor stage (cT stage), and Gleason score obtained on the prostatectomy specimen. Since postoperative determination of a tumor’s Gleason score is superior to the preoperatively determined Gleason score (subjected to sampling errors and consequently under grading in more than one third of cases [8]), this parameter was replaced by the preoperative Gleason score obtained on the original biopsy in scenario 4. High hnRNPA1 expression proved to be an independent prognostic parameter in all scenarios, however, limited to the subset of ERG-negative cancers.

**Table 3:** Multivariate analysis including established prognostic parameters and hnRNPA1 immunostaining

Tumor subset	Scenario	*n* analyzable	*p* value							
			Preoperative PSA-level	pT stage	cT stage	Gleason grade prostatectomy	Gleason grade biopsy	pN stage	R stage	hnRNPA1-expression
All cancers	1	7721	< 0.0001	< 0.0001	–	< 0.0001	–	< 0.0001	< 0.0001	0.0041
	2	11,866	< 0.0001	< 0.0001	–	< 0.0001	–	–	< 0.0001	0.0003
	3	11,673	< 0.0001	–	< 0.0001	< 0.0001	–	–	–	< 0.0001
	4	10,060	< 0.0001	–	< 0.0001	–	< 0.0001	–	–	< 0.0001
ERG-negative cancers	1	3041	0.0001	< 0.0001	–	< 0.0001	–	< 0.0001	0.077	0.0196
	2	4723	< 0.0001	< 0.0001	–	< 0.0001	–	–	0.0008	0.0216
	3	4674	< 0.0001	–	< 0.0001	< 0.0001	–	–	–	0.0028
	4	4598	< 0.0001	–	< 0.0001	–	< 0.0001	–	–	< 0.0001
ERG-positive cancers	1	2420	0.0089	< 0.0001	–	< 0.0001	–	0.0104	< 0.0001	0.0293
	2	3787	< 0.0001	< 0.0001	–	< 0.0001	–	–	< 0.0001	0.1995
	3	3723	< 0.0001	–	< 0.0001	< 0.0001	–	–	–	0.2587
	4	3661	< 0.0001	–	< 0.0001	–	< 0.0001	–	–	0.1664

For definition of the scenarios, see “Statistics” section

## Discussion

The results of our study demonstrate that upregulation of hnRNPA1 is associated with adverse tumor features and poor prognosis in the subset of prostate cancers lacking TMPRSS2:ERG fusions.

That positive hnRNPA1 staining was seen in virtually all cell types of normal prostatic tissue was expected based on its essential and ubiquitous role in the cellular RNA processing machinery [41]. Accordingly, the very small fraction of tissues lacking detectable staining (5%) might first of all indicate technical problems such as under- or overfixation of the donor tissues. Direct comparison of the hnRNPA1 immunostaining intensity in normal and cancerous glands in the same tissue spot indicated that hnRNPA1 becomes upregulated during tumor development. This is an agreement with previous studies. Nadiminty et al. [36] reported higher hnRNPA1 protein and mRNA levels in about 50% of extracts from 27 matched pairs of benign and cancerous prostate samples. Further confirmation comes from publicly available RNA expression data sets (Gene Expression Omnibus GDS1439 [48] and Oncomine “Singh prostate”) showing significantly higher hnRNPA1 mRNA levels in a total of 56 malignant versus 65 benign prostate samples.

We found a strong association between hnRNPA1 upregulation and unfavorable tumor phenotype and adverse clinical outcome in our set of 14,258 interpretable prostate cancers. So far, no comparable IHC studies have been reported. However, overexpression of hnRNPA1 has been previously linked to poor prognosis in hepatocellular carcinoma [50] and breast cancer [37]. Together with our findings, these data argue for a general role of hnRNPA1 for tumor cell aggressiveness. This notion is also supported by data obtained from functional studies. Alternative splicing of cancer-associated genes such as fibroblast growth factor and insulin receptors, signaling kinases such as SRC and RAS, or the BRCA1 tumor suppressor have been reported from hnRNPA1-deregulated cells (reviewed in [17]). In prostate cancer cells, overexpression of hnRNPA1 was associated with increased levels of the androgen receptor splice variant AR-V7 [36], which is constitutively active even in the absence of androgens and has been implicated in the development of the castration-resistant phenotype [15]. A recent study linked overexpression of splicing factors (including hnRNPA1) to hypoxia in PC-3 prostate cancer cells [3], and the authors hypothesized that alternative splicing of cancer-associated genes may help cells to adapt to low oxygen.

The availability of a molecular database from earlier studies using the same TMA or parts thereof enabled us to compare hnRNPA1 expression with other parameters. Here, we included data on the *TMPRSS2:ERG* fusion (occurring in about 50% of prostate cancers), the most common recurrent chromosomal deletions (3p, 5q, 6q, 8p, 10q/PTEN, 12p, 12q, 13q, 16q, 17p, 18q), and the Ki67LI as well as AR protein expression levels. ERG is a member of the ETS family of transcription factors that share a common DNA binding motif [40]. Although ERG overexpression lacks prognostic relevance in prostate cancer [32], it modulates the expression of more than 1600 genes in prostate epithelial cells [18]. The strong association between high hnRNPA1 expression and an ERG-positivity as well as the fact that the hnRNPA1 promoter harbors an ETS binding site [10] suggest that hnRNPA1 belongs to the large group of ERG-regulated genes. One of the many consequences of ERG activation is that it might facilitate epithelial to mesenchymal transition (EMT) in affected cells [39]. Interestingly, high expression of hnRNPA1 has also been shown to promote EMT, at least in gastric cancer [7]. It is, thus, possible that hnRNPA1’s contribution to EMT parallels that of ERG fusion. The complete lack of a prognostic value of hnRNPA1 in ERG-positive cancers, however, might suggest that ERG activation overrides the tumor-promoting function of hnRNPA1. Alternatively, it cannot be excluded that our IHC protocol was not optimally suited to reveal hnRNPA1’s prognostic value specifically in the subset of ERG-positive cancers. In these tumors, hnRNPA1 was so much upregulated that hnRNPA1-negative or weak cancers were virtually absent. A potential prognostic impact could be obscured under these circumstances.

Chromosomal deletions represent the second most frequent type of recurrent genomic aberrations in prostate cancer after *TMPRSS2:ERG* fusions. Their typically large size and heterozygous nature argues for compound haploinsufficiency of multiple affected genes as the underlying mechanism for tumor development and progression [25]. Most deletions in prostate cancer are either linked to an ERG-positive (3p, 8p, PTEN, 12q, 16q, 17q) [21, 24, 25, 28, 29, 49] or ERG-negative status (5q, 6q, 13q, 18q) [6, 20, 23, 26]. It was, thus, not surprising that the ERG-associated hnRNPA1 was linked positively to ERG-associated deletions but inversely to those deletions that prevail in ERG-negative cancers. A search for associations between hnRNPA1 and deletions must, therefore, be restricted to subgroups of ERG-positive and ERG-negative cancers. That virtually all deletions were significantly more frequent in hnRNPA1 overexpressing tumors in our subset analyses strongly suggests a direct or indirect functional link between hnRNPA1 deregulation and genomic instability. Some features of hnRNPA1 may in fact be compatible with a role for genomic instability. hnRNPA1 has been identified as a key factor controlling the LINE-1 DNA element that generates structural DNA alterations when transposed to new genomic locations [11], and functional perturbation of hnRNPA1 has been shown to abrogate the genomic stability and maintenance of telomeres [47].

The strong association of high levels of hnRNPA1 with accelerated cell proliferation, as indicated by a high Ki67LI, fits well to earlier observations linking hnRNPA1 to the translations of cell cycle regulatory genes including fibroblast growth factor, cyclin D1, and cMYC [1]. hnRNPA1 is activated through phosphorylation by Akt and facilitates ribosome entry of mRNA encoding these proteins [19]. A role of hnRNPA1 for cell cycle control is also confirmed in vitro, since knockdown of hnRNPA1 has been shown to inhibit proliferation of lung adenocarcinoma through arrest at the G0/G1 phase of the cell cycle [31]. The Akt dependency of hnRNPA1 activity might also explain the striking link between hnRNPA1 levels and AR expression in our study. Upregulation of the androgen receptor is a well-known consequence of Akt activation in prostate cancer [13].

The Gleason score is the strongest histo-morphological prognostic parameter in prostate cancer. Whereas the “classical” Gleason score in its latest revision defines 5 “prognosis groups” (6, 3 + 4, 4 + 3, 8, 9–10) [9], the quantitative Gleason score provides a more refined estimation of the patient prognosis than classical Gleason grading [42]. That hnRNPA1 expression provided additional prognostic information in patients with Gleason score 4 + 3 cancers, but not in any patient subsets with an identical quantitative Gleason score, demonstrates the power of the quantitative Gleason scoring approach. It is not uncommon that molecular markers can outperform classical, but not quantitative, Gleason grading. In our previous systematic analysis of more than 200 candidate prognosis markers on the same TMA, we found many markers that provide prognostic information beyond classical Gleason scores. In contrast, only few markers, including Centromere protein F [12] and Prostate stem cell antigen [14], were able to beat the quantitative Gleason score at least in one or two of its subgroups. It is intuitive that the sum of all genetic and epigenetic changes in a tumor eventually causes the morphologic changes defining the different Gleason patterns. A single molecular marker may, therefore, not be sufficient to reflect the state of dedifferentiation that is expressed by these morphologic changes. It is possible that future molecular testing will include panels of molecular features, perhaps applied though multiplex fluorescence immunohistochemistry [30]. hnRNPA1 may play a role in such panels, even though its overall prognostic significance was not very strong. This is particularly due to its independent prognostic role in ERG-negative cancers. The limitation of the prognostic impact of hnRNPA1 to a molecular subgroup such as ERG-negative cancers is not an exception. In earlier studies, we had found several prognostic markers that were applicable solely to ERG-negative [5] or ERG-positive cancers [35]. This challenges the concept of developing a prognostic prostate cancer test that is applicable to all tumors.

In summary, hnRNPA1 upregulation is an independent prognosticator for poor disease outcome in ERG-negative prostate cancer. Although its prognostic power is limited as a stand-alone marker, it may be a promising candidate for future multiparametric prognostic tests.

## Electronic supplementary material

ESM 1 (XLSX 11 kb)

ESM 2 (XLSX 11 kb)

ESM 3 (XLSX 10 kb)

ESM 4 (PPTX 40 kb)

ESM 5 (PPTX 12816 kb)

ESM 6 (PPTX 44 kb)
