# Supplementary material for: Upregulation of the heterogeneous nuclear ribonucleoprotein hnRNPA1 is an independent predictor of early biochemical recurrence in TMPRSS2:ERG fusion-negative prostate cancers
Source: Virchows Arch. 2020 May 16;477(5):625–36. doi: 10.1007/s00428-020-02834-4 (PMC7581599; doi:10.1007/s00428-020-02834-4)
Supplement: Supplementary file 4 — (PPTX 40 kb) [file 428_2020_2834_MOESM4_ESM.pptx]

## Slide 1
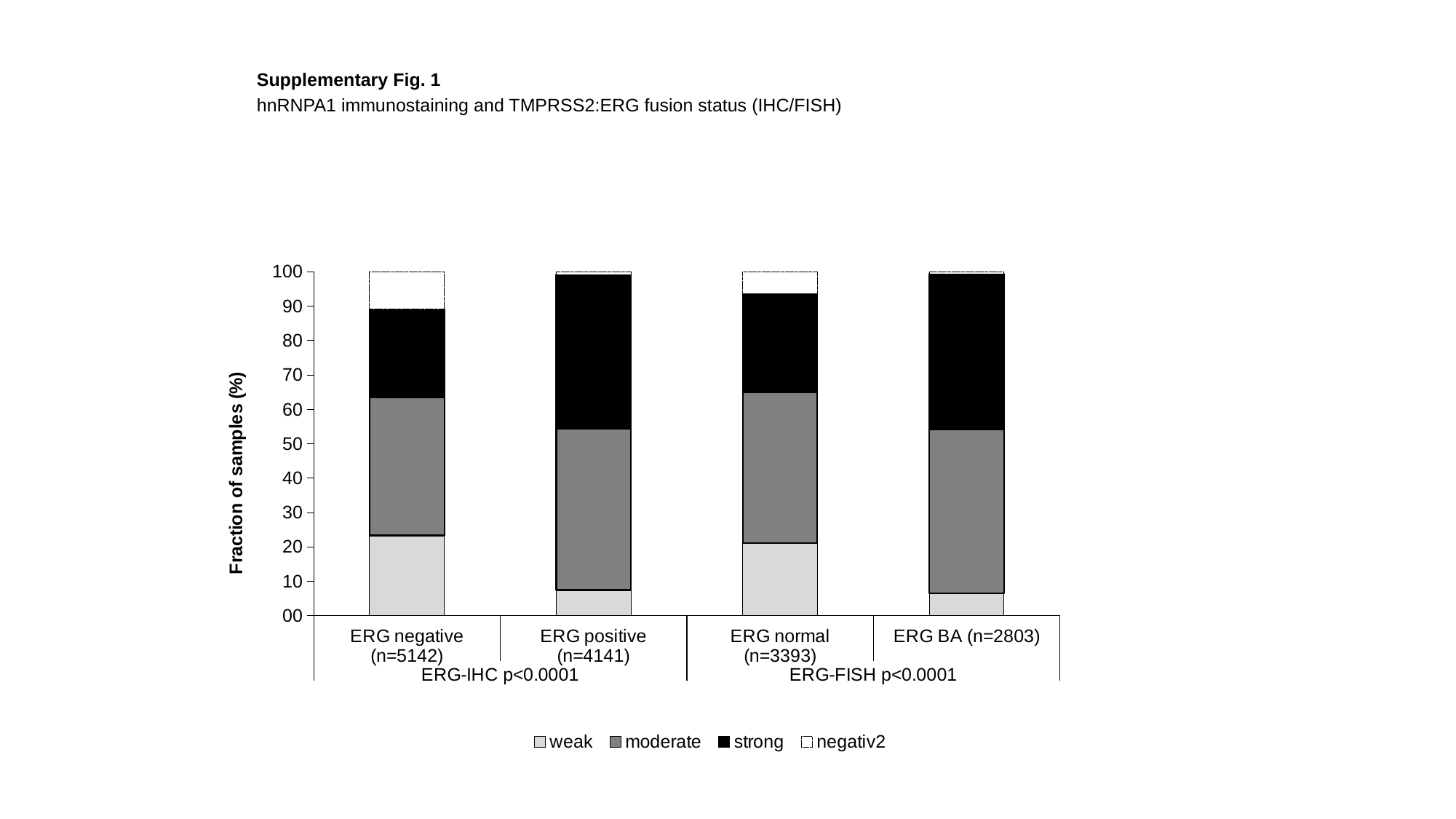

Supplementary Fig. 1
hnRNPA1 immunostaining and TMPRSS2:ERG fusion status (IHC/FISH)
### Chart
| Category | weak | moderate | strong | negativ2 |
|---|---|---|---|---|
| ERG negative (n=5142) | 23.356670556203813 | 40.08168028004667 | 25.670945157526255 | 10.89070400622326 |
| ERG positive (n=4141) | 7.486114465105047 | 46.94518232311036 | 44.74764549625694 | 0.8210577155276504 |
| ERG normal (n=3393) | 21.131741821396993 | 43.9139404656646 | 28.58826996758031 | 6.36604774535809 |
| ERG BA (n=2803) | 6.5643952907599 | 47.59186585800927 | 45.272921869425616 | 0.5708169818052087 |
