# Supplementary material for: Upregulation of the heterogeneous nuclear ribonucleoprotein hnRNPA1 is an independent predictor of early biochemical recurrence in TMPRSS2:ERG fusion-negative prostate cancers
Source: Virchows Arch. 2020 May 16;477(5):625–36. doi: 10.1007/s00428-020-02834-4 (PMC7581599; doi:10.1007/s00428-020-02834-4)
Supplement: Supplementary file 5 — (PPTX 12816 kb) [file 428_2020_2834_MOESM5_ESM.pptx]

## Slide 1
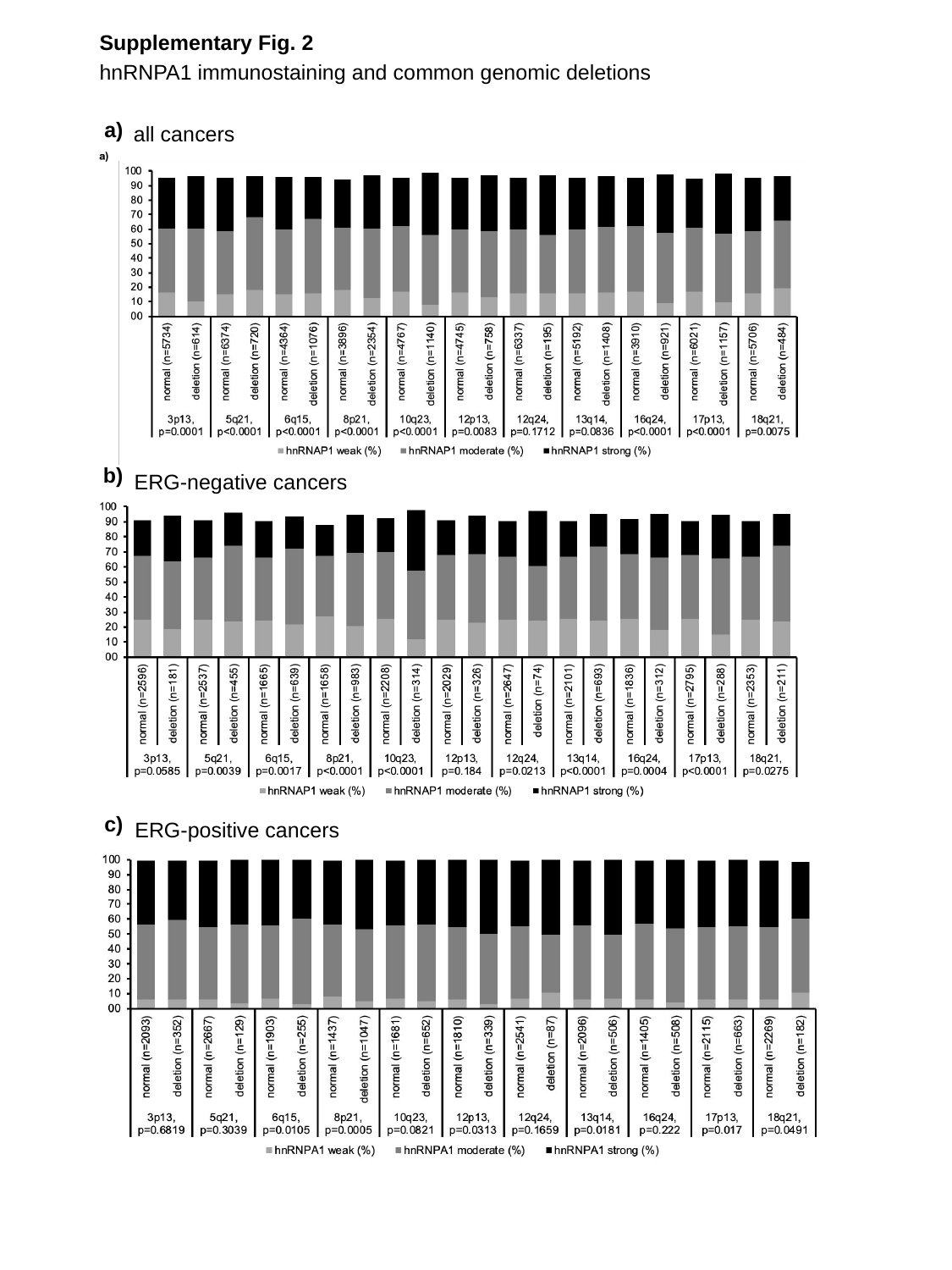

Supplementary Fig. 2
hnRNPA1 immunostaining and common genomic deletions
a)
all cancers
b)
ERG-negative cancers
c)
ERG-positive cancers
