# Supplementary material for: Upregulation of the heterogeneous nuclear ribonucleoprotein hnRNPA1 is an independent predictor of early biochemical recurrence in TMPRSS2:ERG fusion-negative prostate cancers
Source: Virchows Arch. 2020 May 16;477(5):625–36. doi: 10.1007/s00428-020-02834-4 (PMC7581599; doi:10.1007/s00428-020-02834-4)
Supplement: Supplementary file 6 — (PPTX 44 kb) [file 428_2020_2834_MOESM6_ESM.pptx]

## Slide 1
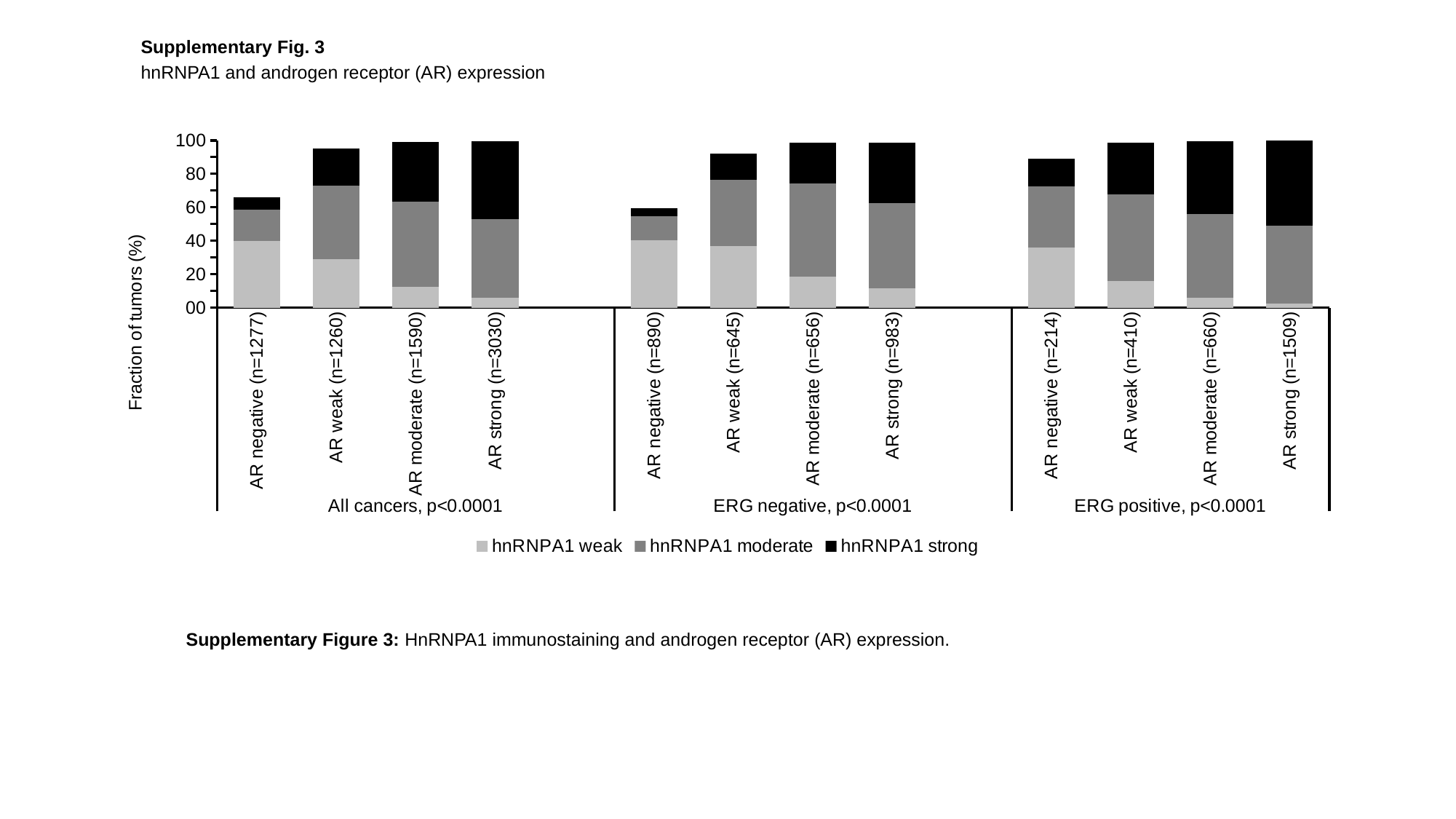

Supplementary Fig. 3
hnRNPA1 and androgen receptor (AR) expression
### Chart
| Category | hnRNPA1 weak | hnRNPA1 moderate | hnRNPA1 strong |
|---|---|---|---|
| AR negative (n=1277) | 39.780736100234925 | 18.872357086922477 | 7.3610023492560686 |
| AR weak (n=1260) | 29.20634920634921 | 43.888888888888886 | 22.063492063492063 |
| AR moderate (n=1590) | 12.38993710691824 | 50.880503144654085 | 35.78616352201258 |
| AR strong (n=3030) | 5.742574257425743 | 47.06270627062706 | 46.6996699669967 |
| | None | None | None |
| AR negative (n=890) | 40.337078651685395 | 14.269662921348313 | 4.831460674157303 |
| AR weak (n=645) | 36.89922480620155 | 39.37984496124031 | 15.968992248062017 |
| AR moderate (n=656) | 18.597560975609756 | 55.487804878048784 | 24.390243902439025 |
| AR strong (n=983) | 11.495422177009155 | 51.06815869786369 | 36.31739572736521 |
| | None | None | None |
| AR negative (n=214) | 35.981308411214954 | 36.44859813084112 | 16.822429906542055 |
| AR weak (n=410) | 15.853658536585366 | 51.951219512195124 | 30.975609756097562 |
| AR moderate (n=660) | 5.757575757575758 | 50.303030303030305 | 43.484848484848484 |
| AR strong (n=1509) | 2.3856858846918487 | 46.52087475149106 | 51.027170311464545 |
Supplementary Figure 3: HnRNPA1 immunostaining and androgen receptor (AR) expression.
